# Dispersion-Corrected Density Functional Theory Investigations of Structural and Electronic Properties of Bulk MoS_2_: Effect of Uniaxial Strain

**DOI:** 10.1186/s11671-015-1099-5

**Published:** 2015-11-04

**Authors:** Chuong V. Nguyen, Nguyen N. Hieu, Duong T. Nguyen

**Affiliations:** Institute of Research and Development, Duy Tan University, K7/25 Quang Trung, Da Nang, Vietnam; School of Mechanical Engineering, Le Quy Don Technical University, Hanoi, Vietnam; School of Engineering Physics, Hanoi University of Science and Technology, Hanoi, Vietnam

**Keywords:** Molybdenum disulphide, Uniaxial strain, Electronic property, Dispersion-corrected density functional

## Abstract

Strain-dependent structural and electronic properties of MoS_2_ materials are investigated using first principles calculations. The structural and electronic band structures of the MoS_2_ with relaxed unit cells are optimized and calculated by the dispersion-corrected density functional theory (DFT-D2). Calculations within the local density approximation (LDA) and GGA using PAW potentials were also performed for specific cases for the purpose of comparison. The effect of strain on the band gap and the dependence of formation energy on strain of MoS_2_ are also studied and discussed using the DFT-D2 method. In bulk MoS_2_, the orbitals shift towards the higher/lower energy area when strain is applied along the *z*/*x* direction, respectively. The energy splitting of Mo4*d* states is in the range from 0 to 2 eV, which is due to the reduction of the electronic band gap of MoS_2_.

## Background

Molybdenum disulphide (MoS_2_) is an interesting material for applications in nanoelectronic applications due to its unique mechanical, electronic, and optical properties [1, 2]. It is a typical layered inorganic material, which is similar to graphite. MoS_2_ triple layers are held together by weak van der Waals (vdW) interactions. It is a typical example of layered transition-metal dichalcogenides family, which has been studied in recent years. The MoS_2_ attracts investigation due its distinctive industrial applications from use as a lubricant [3] and a catalyst [4] as well as in photovoltaics. By chemical bath deposition method and the mechanochemical route, MoS_2_ films have been obtained in the experiments [5]. Due to the interlayer vdW interaction, the bulk MoS_2_ tends to form a bilayer which is known to be an indirect semiconductor. It has indirect energy band gap of 1.23 eV [6]. The bulk MoS_2_ has been used in conventional industries as an intercalation agent and a dry lubricant for many years. In addition, a two-dimensional MoS_2_ is expected for applications in nanoelectronic devices [7].

In recent years, properties of MoS_2_ and its related structures have been theoretically studied [8–13] such as stability of structure, band gap, functionalization through adatom adsorption, and vacancy defects. By means of density functional theory computations, Chen et al. have been systematically investigated the stability and magnetic and electronic properties of MoS_2_ nanoribbons [14, 15]. The defect structure of MoS_2_ has also been studied [16]. Besides, the creation of magnetic and metallic characteristics in low-width MoS_2_ nanoribbons has been studied by the first principles calculations [17]. The electronic structure of MoS_2_ has been also studied[3]. Up to date, many works about MoS_2_ have been done, but some questions are still worth studying. For example, the electronic properties of bulk MoS_2_ under strain have not been enough investigated. Current studies have confirmed that the properties of low-dimensional materials can be modified by strain, therefore, the response of electronic properties of bulk MoS_2_ to the strain would be an interesting issue for discussion.

In the present work, we investigate the strain-dependent structural and electronic properties of low-dimensional MoS_2_ materials using first principles calculations. We apply uniaxial strain onto the bulk MoS_2_. The structural and electronic band structures of MoS_2_ with relaxed unit cells are optimized and calculated by the dispersion-corrected density functional theory (DFT-D2). The effects of strain on the band gap and the dependence of formation energy on strain of MoS_2_ are also studied and discussed.

## Methods

**Fig. 1:**
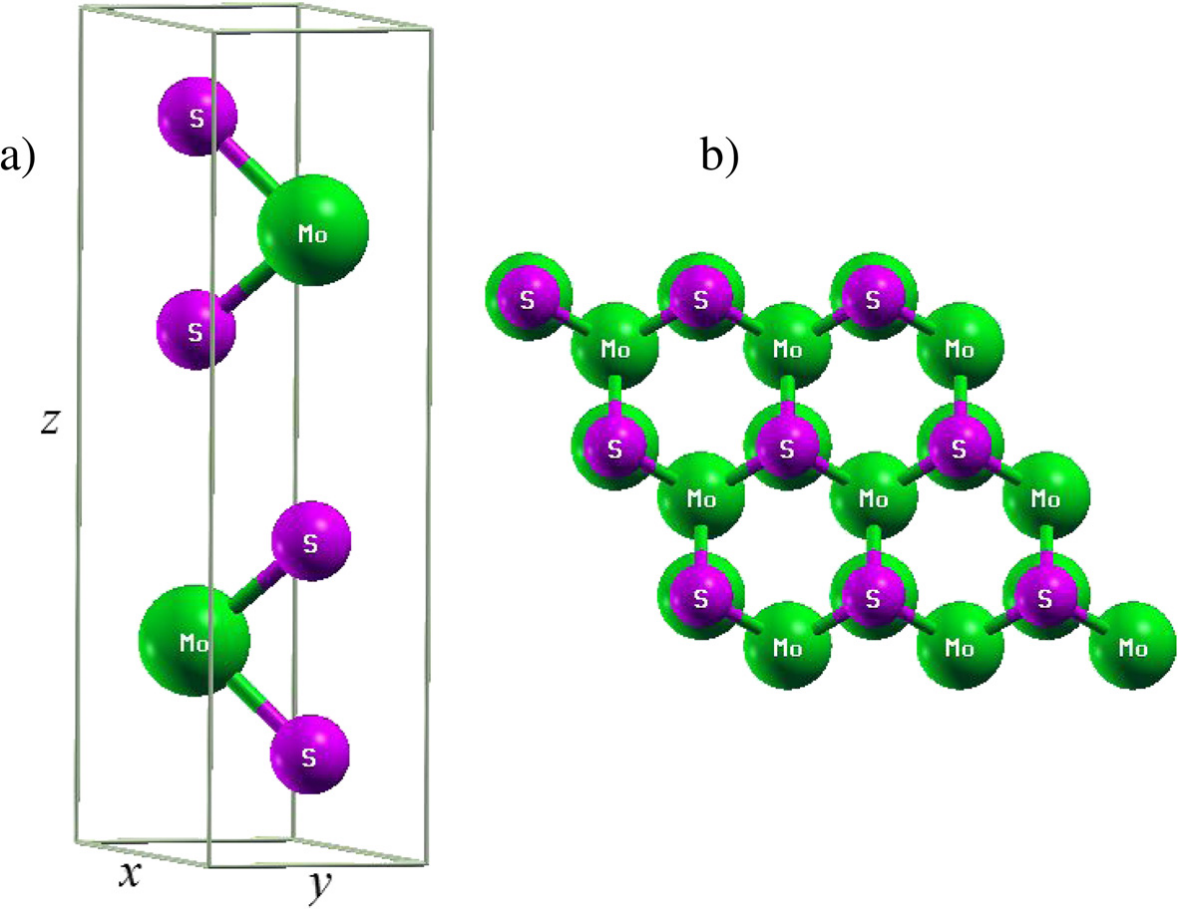
Atomic structure of the bulk MoS_2_. Relaxed atomic structure of the bulk MoS_2_ from DFT-D2 calculations: **a** *top view* and **b** *side view*

The MoS_2_ bulk belongs to the space group P 63/mmc. It is a layered material and a single layer consists of an S-Mo-S sandwich. In each such layer, the Mo atoms are arranged in a hexagonal lattice and are positioned in a trigonal prismatic coordination with respect to the two S layers. This implies that each Mo atom is coordinated by six S atoms. The hexagonal MoS_2_ was selected, and the lattice parameters, *a* = 3.16 Å, *c*/*a* ratio of 0.89 are taken as a starting point for the geometry optimization [6, 18]. Model of bulk MoS_2_ includes two layers of S-Mo-S sandwich, which consist of one Mo atom and two S atoms in each layer, as seen in Fig. 1.

The present calculations are performed within density functional theory using accurate frozen-core full-potential projector augmented-wave (PAW) pseudopotentials [19, 20], as implemented in the Quantum Espresso code [21]. We use the generalized gradient approximation (GGA) with the parametrization of Perdew-Burke-Ernzerhof (PBE) with added van der Waals (vdW) corrections. They are important for describing the interaction between Mo *S*_2_ layers. Beside DFT-D2 method, calculations within the local density approximation (LDA) and GGA using PAW potentials were also performed for specific cases. This combination is for the comparison with DFT-D2 calculations when the vdW interactions were introduced. These vdW interactions were included using the method of Grimme (DFT (PBE)-D2) [22]. This approach has been successful in describing graphene-based structures [23]. For the plane waves used in the expansion of the pseudowave functions, the cutoff energy varies in the range from 450 to 545 eV. The results of the calculations for convergence in the surface energy and interplanar distances confirmed that the cutoff energy of higher than 400 eV and the planar grid with dimensions of 6×6×1 are quite sufficient. For the different layers of the MoS_2_, the supercells are constructed with a vacuum space of 20 Å along the *z* direction. The Brillouin zones are sampled with the *Γ*-centered *K* point grid of 18×18×1. The strain is simulated by setting the lattice parameter to a fixed larger value and relaxing the atomic positions. The magnitude of strain is defined as: *ε*=(*a*−*a*_0_)/*a*_0_, where *a*_0_ and *a* are the lattice parameters of the unstrained and strained systems, respectively.

The traditional density functionals are unable to give a correct description of the vdW interactions because of the dynamical correlations between fluctuating charge distributions. In the present study, we considered the vdW interaction within the DFT framework using a semi-empirical potential via the total energy functional (DFT-D2) as defined by Grimme et al. [22]. This method has been successfully applied for calculations of graphene nanoribbon/h-BN [24–26] and graphene nanoribbon/AlN [27, 28] interfaces. The total energy *E*_tot_ can be expressed as follows [22]:

(1) $$  E_{\text{tot}} = E_{\text{KS--DFT}} + E_{\text{disp}} = E_{\text{KS--DFT}} + E_{\text{vdW}},  $$

where *E*_KS-DFT_ is the usual self-consistent Kohn–Sham energy as obtained from the chosen DFT and *E*_disp_ is an empirical dispersion correction (vdW)

$$E_{\text{disp}} = - \frac{1}{2}\sum_{ij} C_{6ij} \sum_{\mathbf{R}}\left|\mathbf{r}_{ij} + \mathbf{R}_{ij}\right|^{-6} f_{\text{damp}}\left(| \mathbf{r}_{ij}+ \mathbf{R}_{ij}|\right) $$

and

$$f_{\text{damp}}\left(| \mathbf{r}_{ij}+ \mathbf{R}_{ij}|\right) \!= s_{6}\! \left\{1 + \exp\! \left[ -d\!\left(\frac{| \mathbf{r}_{ij} + \mathbf{R}_{ij}|}{r_{0}} - 1\! \right) \!\right] \right\}^{-1}, $$

where **r**_*ij*_=(**r**_*j*_−**r**_*i*_) is the atom–atom distance vector, **R**=*l***a**+*m***b**+*n***c** is the lattice vector, *s*_6_ is the functional-dependent scaling parameter, and *d* is a parameter that tunes the steepness of the damping function (*d*=20, *s*_6_=0.75 for PBE). The *C*_6*ij*_ coefficients are computed for each atom pair by the geometric mean of atomic terms ${C_{6ij}} = \sqrt {{C_{6i}}.{C_{6j}}} $, and the *r*_0_ term is computed by the simple sum of vdW radii of the atom pairs *r*_0_=*r*_0*i*_+*r*_0*j*_.

## Results and Discussion

### Structural and Electronic Properties of the Bulk MoS_2_

Our calculations for the geometrical parameters and band gap of the bulk MoS_2_ using different methods are listed in Table 1. By using three different methods, our calculations show that the lattice parameter *a* for the bulk MoS_2_ is 3.116, 3.172, and 3.176 Å corresponding to the LDA, GGA, and DFT-D2 methods, respectively. This result is in good agreement with other theoretical [29, 30] and experimental [6, 18] studies, as shown in Table 1.

**Table 1:** Calculated structural parameters and band gap of the bulk MoS_2_ using LDA, GGA, and DFT-D2 methods

	Lattice constant		*E*_*g*_, eV	*d*_*Mo*−*S*_, eV
	*a*, Å	*c*/*a*		
LDA	3.115	3.85	0.72	2.36
GGA	3.172	3.95	0.96	2.43
DFT-D2	3.176	3.85	1.22	2.41
Theory (LDA)	3.13 [29]	3.84 [29]	0.75 [29]	2.39 [29]
	3.11 [30]	-	0.72 [30]	2.37 [30]
Theory (GGA)	3.23 [29]	4.01 [29]	1.05 [29]	2.45 [29]
	3.20 [30]	-	0.85 [30]	2.42 [30]
Experiment	3.16 [18]	3.89 [18]	1.23 [6]	2.41 [18]

We calculate the electronic band gap of the bulk MoS_2_ by using different methods (LDA, GGA, and DFT-D2). We see that the band gap value calculated by the LDA and GGA methods is smaller than that of the experimental study. Our result for the band gap of the bulk MoS_2_ form is 0.72 eV (0.96 eV) using LDA (GGA) functionals, which is in good agreement with the available theoretical data of 0.72 eV (0.85 ev) using the same LDA-PAW (GGA) funtionals [30, 31]. These values are smaller than that of the experimental study (1.23 eV) [6]. This difference is due to the inherent drawback of standard LDA/GGA functionals. However, the DFT-D2 calculations for the band gap give results (1.20 eV) that are in good agreement with the experimental data (1.23 eV) [6]. Besides, our DFT-D2 calculations give the bond length *d*_*Mo*−*S*_ being 1.41 Å. That is the same value as in the experimental study. The match between the DFT-D2 method and the experimental study can be explained by the existence of the vdW interaction in MoS_2_. Our DFT-D2 calculations is including the vdW interaction. We believe that the DFT-D2 method is a suitable method for the structural and electronic properties of the bulk MoS_2_.

**Fig. 2:**
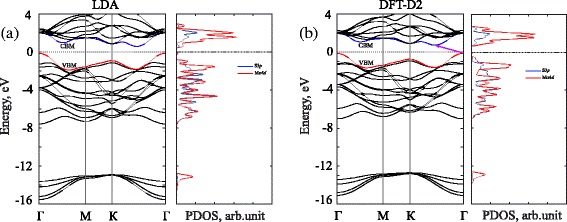
Electronic band structure of the bulk MoS_2_. Band structure and projected density of state (PDOS) of the bulk MoS_2_ by using LDA (**a**) and DFT-D2 (**b**) methods. The Fermi energy was set to zero

Figure 2 shows the projected density of state (PDOS) of the ideal bulk MoS_2_ form. It shows that the valence band consists of two main bands, in which the lower band (from −14.0 eV to −13.0 eV) below the Fermi level *E*_*F*_ (*E*_*F*_=0) is mainly due to S3*s* states. Upper valence bands (from −7.0 eV to −0.5 eV) involve the noticeable contributions of the Mo4*d* and S3*p* states. This results in hybridized Mo4*d*-S3*p* interactions and provides the covalent component of the Mo-S bonds in bulk MoS_2_. Besides, the lower edge of the conduction band of the bulk MoS_2_ involves mainly the antibonding Mo4*d* states. The bands around the energy band gap are relatively flat, as expected from the *d* character of the electron states at these energies. Our DFT-D2 band structure calculations show that the bulk MoS_2_ has an indirect band gap of 1.22 eV opening between the lowest energy of the conduction band (located at between the *Γ* and *K* points) and the highest energy of the valence band (located at the *Γ* point) (see Fig. 2).

### Effect of Uniaxial Strain on the Structural and Electronic Properties of MoS_2_

In this part, we consider the influence of uniaxial strain on the structural and electronic properties of the bulk MoS_2_. Uniaxial strain along both *x* and *z* directions is considered in our study (see, Fig. 1). The components of strain along the *x* and *z* directions are noted as *ε*_*x*_ and *ε*_*z*_, respectively. The strains are evaluated as the lattice stretching percentage. We defined *ε*_*x*_=(*a*−*a*_0_)/*a*_0_ and *ε*_*z*_=(*c*−*c*_0_)/*c*_0_, where *a*_0_ and *c*_0_ are the lattice constants at the equilibrium state, and *a* and *c* are strained lattice constants. A wide range of strain along (up to 10 *%*) both directions with step *Δε*=2 *%* has been employed in the present study.

**Fig. 3:**
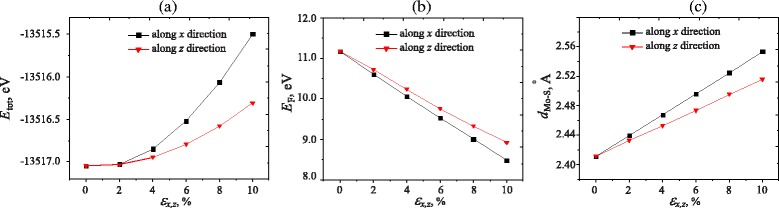
MoS_2_ under uniaxial strain. Dependence of the total energy (**a**), Fermi energy (**b**), and bond length *d*_*Mo*−*S*_ (**c**) on the strain along both *x* and *z* directions

Figure 3 shows the dependence of the total energy, the Fermi energy *E*_*F*_, and bond length *d*_*Mo*−*S*_ on the strain. At the equilibrium state (unstrained), the total energy of the bulk MoS_2_ is minimum. The dependence of total energy on uniaxial strain can be described by a hyperbolic shape as shown in Fig. 3a. Figure 3a also shows that the total energy of bulk MoS_2_ along the *x* direction is higher than that along the *z* direction. When the strain along the *x* direction is applied, the bulk MoS_2_ turns out to be less stable than that of the strain along the *z* direction.

Figure 3b, c shows the dependence of Fermi level *E*_*F*_ and bond length *d*_*Mo*−*S*_ in the bulk MoS_2_ on the strain along *x* and *z* directions. Under uniaxial strain, the Fermi energy and the Mo-S bond length change linearly with strain. As the uniaxial strain increases, the Fermi energy is decreased and the bond length *d*_*Mo*−*S*_ increases. We can see that the total energy, Fermi energy, and bond length depend not only on strain strength but also depend strongly on the direction of the applied strain.

**Fig. 4:**
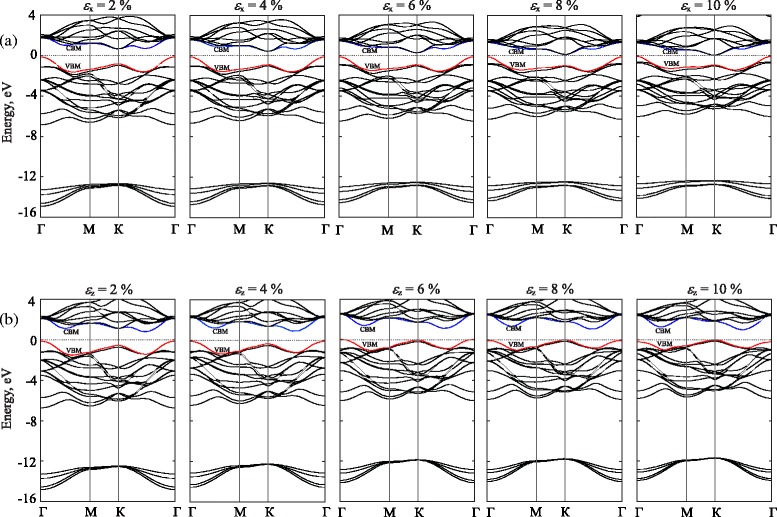
Band structure of the bulk MoS_2_ under uniaxial strain. The applied strain is along the *x* axis (**a**) and along *z* axis (**b**). The *blue* and *red lines* stand for the lowest conduction (CBM) and highest valence (VBM) subbands, respectively. For comparison, the energies are aligned with respect to the valence band top at the *Γ* point

**Fig. 5:**
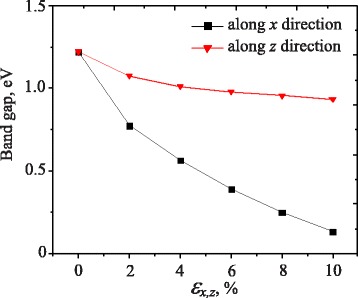
Dependence of the MoS_2_ band gap on uniaxial strain

The effect of the uniaxial strain on the electronic band structure and energy band gap of bulk MoS_2_ is shown in Figs. 4 and 5, respectively. Both *x* and *z* directions of strain are taken into account. We can see that the electronic properties of bulk MoS_2_ are sensitive to the uniaxial strain. The band gap strongly depends not only on the elongation but also on the strain direction (see, Fig. 5). As shown in Fig. 4, we can see that in the bulk MoS_2_, the orbitals will be shifted towards a higher/lower energy level at the *K* point when strain is applied along the *z*/*x* direction, respectively. The states at top of the valence band and bottom of the conduction band near the *Γ* point, which originate mainly from *d* orbitals on Mo atoms and contributions of *p*_*z*_ orbitals on S atoms, are accordingly independent on the uniaxial strain. When *ε*_*x*_ strain is applied, we observe a shift of orbitals in the conduction bands towards the lower energy region, while all orbitals of valence bands shift toward the Fermi level, which is in result of a reduction of the band gap energy. Similarly, when *ε*_*z*_ strain is applied, the shift of orbitals are also observed but in the valence band. It shifts towards the higher energy region.

At equilibrium, the lowest energy of the conduction band $\left (E_{K}^{\text {CBM}}\right)$ and the highest energy of the valence band $\left (E_{K}^{\text {VBM}}\right)$ are 0.903 and −0.736 eV, respectively. These values of energy are decreased due to increasing the strain strength. Especially, when the strain increases from 0 to 10 *%*, $E_{K}^{\text {CBM}}$ decreases from 0.903 to 0.08 eV.

Figure 5 depicts the energy band gap as a function of the applied *ε*_*xz*_ strain. Under *ε*_*x*_ strain, the band gap decreases monotonically with strain. We can see that at *ε*_*x*_=2 *%*, the band gap is equal to 0.78 eV. This band gap decreases to 0.13 eV when *ε*_*x*_=10 *%*. The effect of the *ε*_*z*_ on the band gap of the MoS_2_ is negligible (in comparison to the case of strain applied along the *x* direction). We see that when the *ε*_*z*_<6 *%*, the point in *k*-space corresponding to the highest energy of the valence band is located at the *Γ* point and it will be shifted to the *K* point in the first Brillouin zone when the *ε*_*z*_>6 *%*. The band gap of the bulk MoS_2_ is strongly dependent on the applied strain along the *x* direction and we expect that a phase transition will occur in the case of larger deformation.

In addition, we also calculate band gap of bulk MoS_2_ under uniaxial strain along the armchair direction (*y* direction). Similar to the case of strain along the zigzag direction, band gap of MoS_2_ reduces concurrently with strain. Our calculations show that the change in band gap of MoS_2_ under uniaxial strain along the zigzag and armchair directions is almost the same. This result is in good agreement with the previous works [32, 33].

**Fig. 6:**
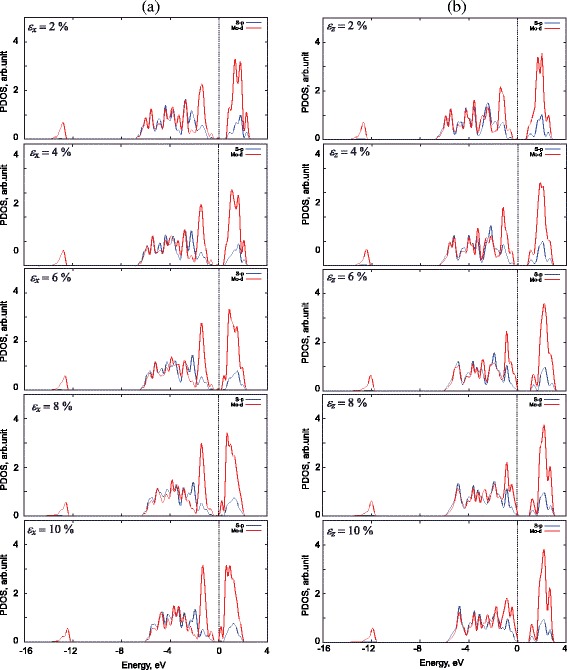
The PDOS of the Mo4*d* and S3*p* states under strain. The PDOS of the Mo4*d* and S3*p* states of the bulk MoS_2_ form under applied *ε*_*x*_ (**a**) and *ε*_*z*_ (**b**) strain, respectively

**Fig. 7:**
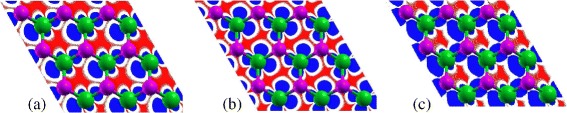
Isosurfaces of lowest unoccupied crystal orbitals (CBM) at the *K* point in the first Brillouin zone of the bulk MoS_2_ under the applied strain. **a** *ε*_*xz*_=0, **b** *ε*_*x*_=8 *%*, **c** *ε*_*z*_=8 *%*

The strain dependence of the projected density of states (PDOS) of the Mo4*d* and S3*p* states is shown in Fig. 6. It shows that, under the uniaxial strain, an energy splitting of Mo4*d* states in the range from 0 to 2 eV is observed. The effect may be associated with crystal field theory, in which the loss of degeneracy of Mo4*d* orbitals in transition metal complexes is described. The energy splitting of the Mo4*d* orbitals is observed in both cases of strain, as shown in Fig. 6. Figure 7 shows the isosurface of lowest unoccupied crystal orbitals at the *K* point in the first Brillouin zone of the bulk MoS_2_ in the case of with and without strain. Based on the partial charge density distribution, we see that the change in electronic properties of the bulk MoS_2_ is determined by the strength of the Mo-S bond. Figure 7 also shows that the lowest energy of the conduction band at *K* point is mainly contributed by the coupling between the Mo4*d* and S3*p* orbitals.

## Conclusions

In this paper, we studied the effect of uniaxial strain on the structural and electronic properties of the bulk MoS_2_ using first principles calculations. Methodologically, we pointed out that the DFT-D2 calculations are a suitable method for calculations of structural and electronic properties of the bulk MoS_2_. Our calculations showed that the electronic properties of the bulk MoS_2_ are very sensitive to the uniaxial strain, especially when the strain is applied along the *x* direction. The band gap of the bulk MoS_2_ decreases linearly with an increase of the strain strength and we can control the energy splitting and band gap of the bulk MoS_2_ by the strain. This makes MoS_2_ becoming a promising material for application in nanoelectronic device such as nanosensors.
